# Unité mobile de soins pour la prise en charge d’infections à transmission aérienne

**DOI:** 10.48327/mtsi.v2i2.2022.232

**Published:** 2022-04-08

**Authors:** Pierre Gazin

**Affiliations:** SFMTSI Société francophone de médecine tropicale et santé internationale (ancienne SPE), Hôpital Pitié-Salpêtrière, Pavillon Laveran, 47-83 Boulevard de l’Hôpital, 75651 Paris cedex 13, France

**Keywords:** Unité mobile de soins, Épidémie, Infection, Transmission aérienne, Pression négative, Croix-Rouge, Mobile care unit, Outbreak, Infectious disease, Airborne transmission, Negative pressure, Red Cross

## Abstract

Une unité mobile de soins constituée de tentes a été conçue pour la prise en charge des malades infectieux. Elle dispose de pression aérienne négative. Indications et contraintes d’emploi de ce prototype, qui n’a pas encore été expérimenté sur le terrain, sont présentées.

## Abstract (en)

A mobile care unit has been designed for infectious patients care in a humanitarian setting. Six individual compartments are grouped together in the main tent of 54 m^2^ under negative air pressure. The principle of walking from clean to dirty is respected. The main indications are bacterial or viral infections with airborne transmission and an epidemic potential. The operating situations of this prototype, which has not yet been tested in the field, and its constraints in an underdeveloped country are presented.

## Introduction

Le mouvement Croix-Rouge dispose d’équipes et de structures prévues pour répondre à des situations d’urgence dans des pays tiers, hors conflit armé [1]. Le déploiement de ces Équipes de Réponse aux Urgences (ERU, plus connues comme *Emergency Response Unit*) aux compétences diverses et complémentaires est décidé et coordonné par la Fédération internationale des sociétés de la Croix-Rouge et du Croissant Rouge pour des interventions de courte durée, ne dépassant pas en principe quatre mois éventuellement renouvelés, en appui à des structures nationales insuffisantes. La Croix-Rouge française dispose ainsi d’une équipe WASH chargée de la distribution d’eau, de la gestion des eaux usées et de la promotion de l’hygiène, d’une équipe *Relief* chargée de fournir des équipements de base aux victimes et d’une équipe Santé. Cette dernière a pour mission d’offrir des soins de base curatifs et préventifs en ambulatoire ou avec une courte surveillance ainsi que de participer à la formation de professionnels de santé du pays d’accueil. Ces équipes ont été confrontées à des épidémies de maladies infectieuses (choléra en Haïti entre 2010 et 2016, maladie à virus Ebola en Guinée Conakry de 2013 à 2016 et en République démocratique du Congo en 2018 et 2020) ce qui les a incitées à concevoir en collaboration avec la société UTILIS^1^ une unité mobile de soins adaptée à la prise en charge d’infections épidémiques.

## Présentation de l’unité mobile à pression négative

**Figure 1:**
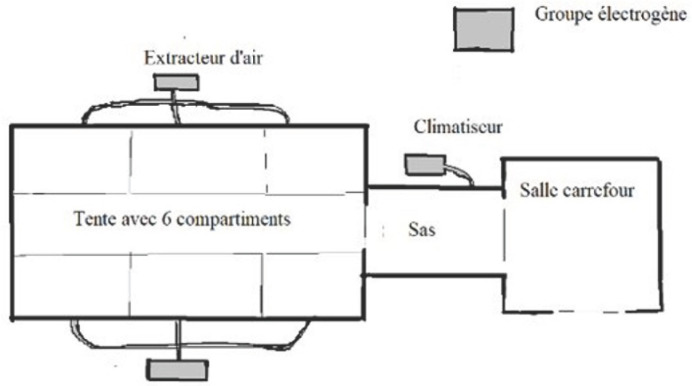
Plan simplifié de l’unité mobile de soins Simplified plan of the mobile care unit

Ce prototype se compose d’une tente de 54 m^2^ avec 6 compartiments individuels pouvant être maintenus en pression négative (≈ -25 kPa), un sas de 10 m^2^ et une pièce « carrefour » de 20 m^2^ en pression normale (Fig. 1 et 2). Ce sont des tentes en toile plastique aux propriétés virucides sur les deux faces^2^, soutenues par une charpente externe avec haubanage, disposant d’un plancher rigide et d’un filet thermique protecteur. L’aspiration continue est assurée par un extracteur (3 000 m^3^/heure) (Fig. 3). L’air rejeté est filtré au moyen d’un filtre Très Haute Efficacité classe H14 (rétention des gouttelettes aérosol de 0,3 μm). Un climatiseur de 23 kW permet le maintien d’une température acceptable dans le sas et la zone infectieuse. L’autonomie énergétique est assurée par un groupe générateur de 33 kVA. Les compartiments individuels disposent d’un lit et de toilettes sèches avec traitement chimique des excreta. Leur paroi donnant sur le couloir central est transparente et peut être occultée pour préserver l’intimité. Des sas permettent l’apport de nourriture et de traitements. Le personnel n’est ainsi pas obligé d’être en tenue EPI complète tant qu’il ne pénètre pas dans les compartiments. L’ensemble du dispositif est stocké dans 18 containers réutilisables de 2,1 x 1 x 0,9 m (Fig. 4).

**Figure 2:**
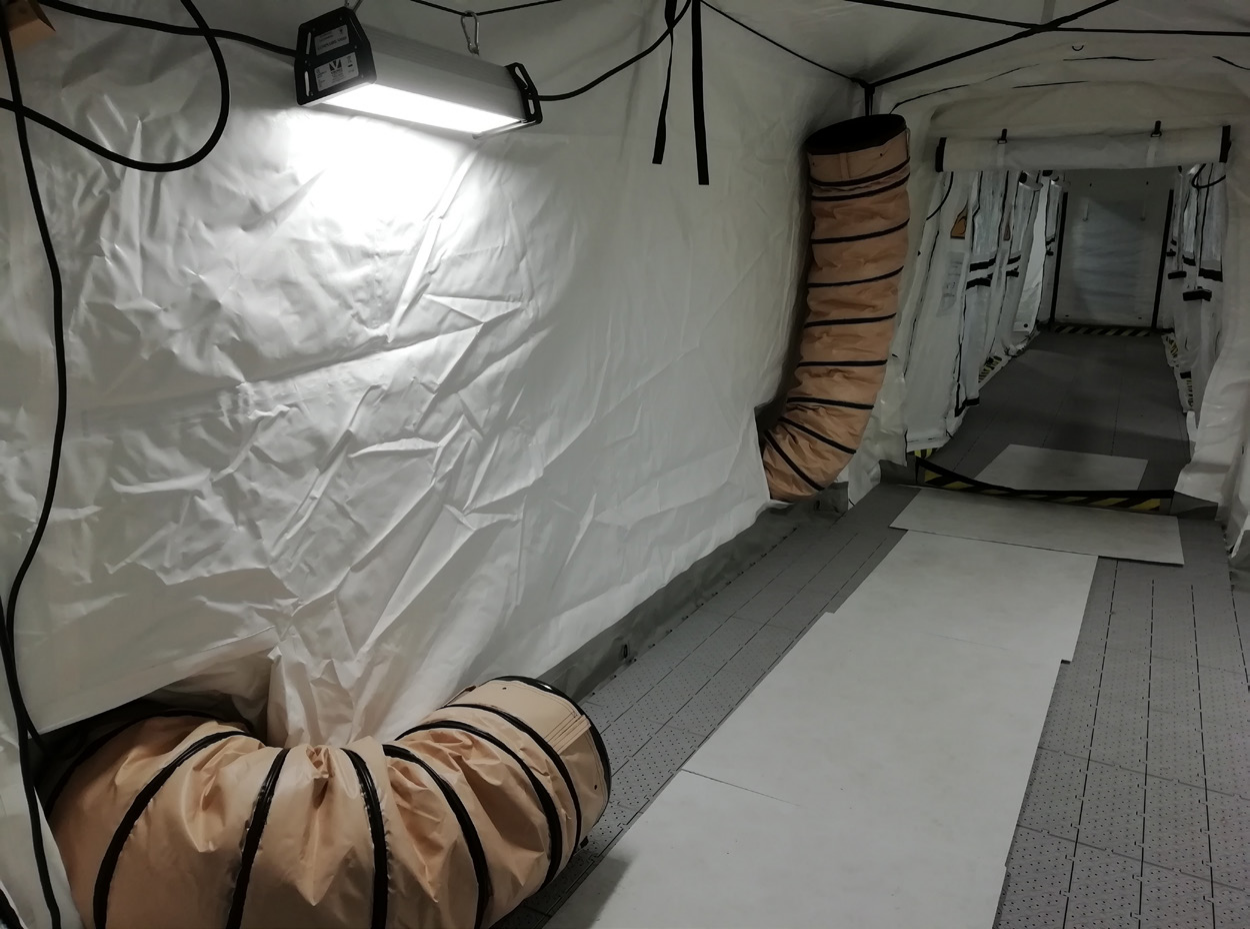
Intérieur du sas et de la tente en pression négative Interior of the transfer airlock and tent in negative pressure

**Figure 3:**
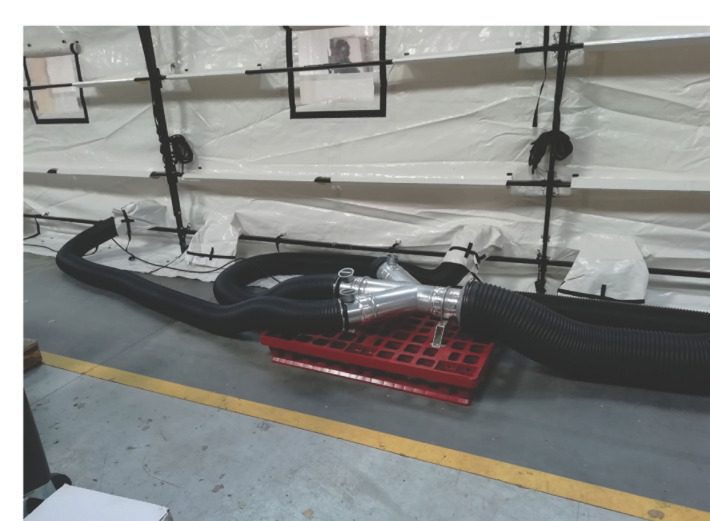
Collecte de l’air pour l’aspiration Pipes for air suction

**Figure 4:**
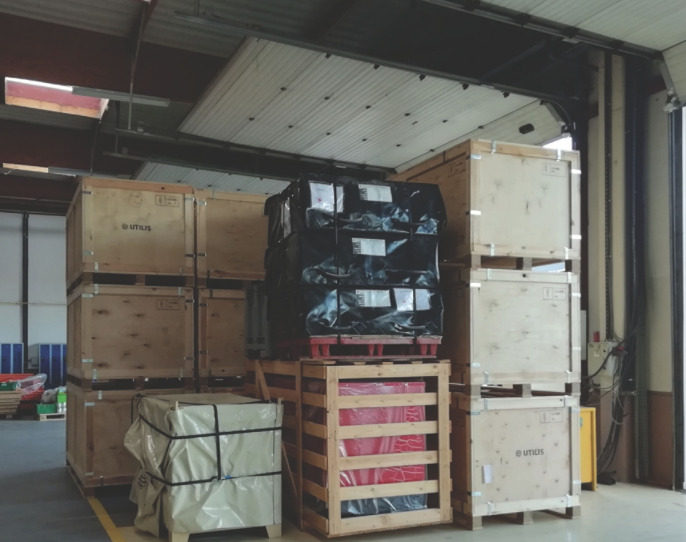
Ensemble du matériel rangé avant son transport All equipment stored before transport

## Pour quelles pathologies?

Cette structure est conçue pour traiter jusqu’à 6 malades contagieux gardés en soins et surveillance pendant quelques jours. Quelles pathologies peuvent-elles être concernées?

- Le choléra, de transmission essentiellement oro-fécale, nécessitant des soins intenses de réhydratation et de toilette, ne concerne pas cette structure.
- La peste peut avoir une transmission aérienne. La pression négative est donc utile.
- Les fièvres hémorragiques virales telles que les fièvres de Lassa, Marburg, Ebola. La transmission est essentiellement par un contact avec les fluides des malades. La pression aérienne négative est une précaution supplémentaire, mais non essentielle.
- Les infections par des virus à transmission aérienne: virus de la grippe et leurs nombreux variants, virus du SRAS, du MERS, de la Covid-19. La pression négative prend alors tout son intérêt.
- La fièvre Q avec sa possible transmission aérienne.
- La tuberculose pulmonaire éventuellement, bien que sa prise en charge sorte du cadre d’une réponse urgente à une épidémie.
- Le monkeypox ou variole du singe et sa possible transmission interhumaine par voie aérienne.

## Les contraintes

Les contraintes pour la mise en place et le bon fonctionnement de cette unité mobile sont importantes:

- Acceptation par les autorités sanitaires du pays bénéficiaire. Une structure provisoire n’est pas toujours acceptée par des autorités qui peuvent être davantage dans la demande de structures pérennes.
- Disponibilité d’un terrain sécurisé et d’un accès à l’eau. La structure nécessite un terrain d’au moins 500 m^2^, plat, bien drainé, disposant d’un accès à l’eau, clos et sécurisé en particulier la nuit.
- Acceptation par la population environnante. Une unité de soins de maladies infectieuses peut être mal acceptée car soupçonnée de favoriser la diffusion de l’agent infectieux, voire de son introduction.
- Financement et transport. Hors matériel médical, la structure, y compris le générateur électrique, pèse environ quatre tonnes. Son coût, en incluant son transport aérien, est de l’ordre de 250 000 euros. Dans la grande majorité des cas, le matériel envoyé sur le terrain y est laissé en fin de mission.
- Capacité à travailler avec les acteurs locaux de la santé avec des moyens techniques correspondant aux normes du pays, en gardant en tête les dangers de la substitution et du court terme.
- Capacité à attirer les malades. Un refus de l’hospitalisation peut survenir, favorisé par les inévitables rumeurs et désordres engendrés par l’épidémie. L’unité de soins peut être perçue comme dangereuse ou comme un inutile mouroir.
- Nécessité de prévoir dès le début des activités la fin de celles-ci, leur transfert à des acteurs locaux de la santé en favorisant leur intégration à l’équipe soignante, leur éventuelle formation, en organisant l’apport de consommables et sa continuité.
- Maintenance, en particulier entretien régulier des filtres de l’extracteur selon un protocole adapté à ce matériel à haut risque infectieux.

## Discussion

**Figure 5:**
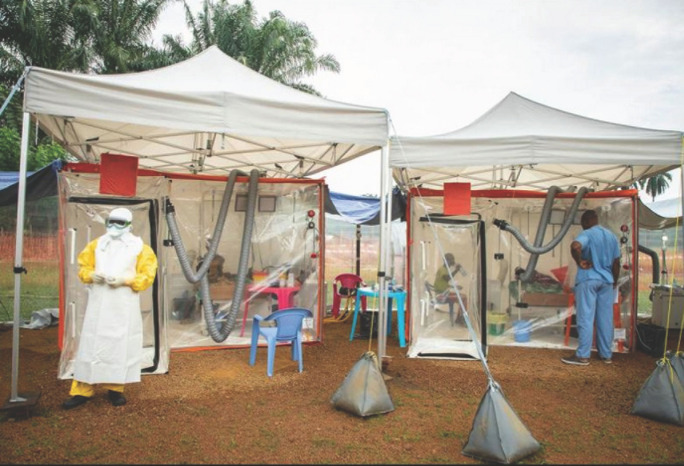
Structures CUBE pour la prise en charge de maladie à virus Ebola en RDC en 2018 CUBE structures for Ebola virus disease care in DRC in 2018

Ce prototype d’unité mobile de soins est conçu pour être utilisé dans des zones souffrant d’un déficit de moyens pour la prise en charge d’épidémies, avec un centrage sur les pathogènes à transmission aérienne. Ses qualités et ses défauts restent à découvrir, car cette unité n’a encore jamais été utilisée sur le terrain. Cependant, une expérience importante a déjà été acquise avec le CUBE (Chambre d’urgence biosécurisée pour épidémies) déployé par l’ONG Alima à Beni (RDC) en 2018 dans le cadre d’une épidémie de fièvre à virus Ebola [2]. En comparaison avec les premiers centres de traitement Ebola en Guinée, ces structures individuelles ont permis un considérable allégement des charges de travail du personnel soignant et une ambiance bien moins anxiogène pour les malades et leurs proches (Fig. 5). Ces structures séparées les unes des autres facilitent le contact des malades avec l’extérieur, évitant ainsi le sentiment d’isolement, ce qui est important pour des durées moyennes de séjour de 16 jours. Le maintien des contacts avec l’entourage paraît plus difficile dans la structure présentée ici avec son couloir unique limité par un sas et situé au centre des deux rangées de trois compartiments. Le coût du CUBE est également élevé et les contraintes logistiques (eau, électricité, sécurité) sont équivalentes à celle du prototype présenté. La perception de la maladie, du risque, du pronostic, l’acceptation des soins dépendent en grande partie des conditions d’accueil des malades dans la structure de soins. L’expérience acquise en Guinée en 2015 dans le Centre de traitement des soignants mérite d’être prise en compte [3].

Une alternative à ces structures mobiles est la construction en semi-dur (dalle en ciment, bas des murs en briques, haut en bois, couverture en tôle) telle que celle réalisée à Mbandaka (RDC) en 2020. Le coût en est probablement moindre et la durabilité plus importante. Cependant, la réalisation prend davantage de temps. Dans l’idéal, les structures mobiles et en semi-dur doivent être envisagées comme complémentaires.

## Conclusion

Cette unité mobile pourra se révéler utile dans des situations d’urgence à condition de disposer d’un budget adéquat et de prévoir dès sa mise en place le passage à une structure plus pérenne. L’actuelle pandémie de Covid-19 pourrait amener à l’utiliser là où les moyens locaux seraient dépassés, y compris dans les pays les plus développés.

## Liens d’intérêts

P. Gazin est un équipier ERU de la Croix Rouge.
